# Impact of Surgical Margin Distance on Oncologic Outcomes in Vulvar Squamous Cell Carcinoma

**DOI:** 10.3390/jcm14124057

**Published:** 2025-06-08

**Authors:** Caroline Lenz, Su Ir Lyu, Peter Mallmann, Bernd Morgenstern, Fabinshy Thangarajah

**Affiliations:** 1 Department of Gynecology and Gynecologic Oncology, Center for Integrated Oncology Aachen Bonn Cologne Düsseldorf, University Hospital of Cologne, 50931 Cologne, Germany; caroline.lenz@uk-koeln.de (C.L.); kontakt@gynpraxis-mallmann.de (P.M.); bernd.morgenstern@uk-koeln.de (B.M.); 2 Department of Pathology, University Hospital of Cologne, 50931 Cologne, Germany; su.lyu@uk-koeln.de; 3 Department of Gynaecology and Obstetrics, University Hospital of Essen, 45147 Essen, Germany

**Keywords:** vulva carcinoma, margin distance, surgical treatment, recurrence

## Abstract

**Introduction:** Vulvar carcinoma is a rare malignancy with approximately 3090 cases annually in Germany. Squamous cell carcinoma of the vulva (SCC) can be HPV-associated or non-HPV-associated. Surgical excision is the primary treatment, but the optimal tumor-free resection margin remains debated. This study evaluates the impact of resection margin distance on disease prognosis in SCC patients. **Methods:** A retrospective analysis was conducted on patients diagnosed with SCC of the vulva at the University Hospital of Cologne between 2007 and 2022. Patients with TNM stage pT1a or higher who underwent primary surgical treatment with a tumor-free resection margin status were included. The cohort of 73 patients was divided into three groups based on resection margin distance: >0.01–<0.3 cm (Group 1), ≥0.3–<0.8 cm (Group 2), and ≥0.8 cm (Group 3). Statistical analyses including logrank tests were performed to assess correlations between resection margin distance and recurrence, disease-free survival (DFS), and overall survival (OS). **Results:** A total of 37.0% of patients were categorized into Group 1, 48.0% into Group 2, and 15.1% into Group 3. Disease recurrence occurred in 26% of patients, with rates of 31.6% in Group 1 (minimum resection margin distance), 42.1% in Group 2, and 26.3% in Group 3. The prevalence of recurrence was not dependent on resection margin distance (*p* = 0.28). Similarly, the prevalence of deaths was not dependent on resection margin distance (*p* = 0.43). Disease-free survival (DFS) and overall survival (OS) did not show significant differences between the groups (*p* = 0.25 and *p* = 0.87, respectively). **Conclusions:** This study found no significant impact of pathological tumor-free resection margin distance on DFS or OS in patients with SCC of the vulva. Given the heterogeneity in international guidelines and existing literature, further studies with larger patient cohorts are needed to establish definitive surgical recommendations.

## 1. Introduction

With an incidence of 3090, carcinoma of the vulva is the fifth most common gynecological malignancy in women. With a median age of onset of 73 years, primarily affecting older women [1,2,3,4]. Most cases are diagnosed at an early stage, contributing to a 5-year survival rate of 70%. More than 90% of vulvar carcinomas are squamous cell carcinomas (VSCC). On a molecular level, classification has evolved beyond simply distinguishing between HPV-associated and HPV-independent carcinomas. It now also includes p53 status. Consequently, VSCC can be prognostically subdivided into three groups: HPV-associated (HPV+), HPV-independent/p53 wild type (HPV-/p53wt), and HPV-independent/p53 abnormal (HPV-/p53abn) [5,6,7]. In the early stages, surgery remains the first-line therapy [8].

Over recent decades, radical vulvectomy and systematic lymphadenectomy have been largely replaced by wide radical excision with or without lymph node assessment, depending on the depth of tumor infiltration [9,10]. In advanced vulvar carcinoma, radiotherapy or cisplatin-based chemoradiotherapy could be considered to reduce surgical morbidity or as a neoadjuvant treatment to avoid exenteration. Adjuvant radiotherapy should be performed by modern intensity-modulated radiotherapy techniques (IMRT/VMAT-like) [8,11]. Due to the anatomical proximity of critical structures such as urethra, clitoris, and anus, surgery for vulvar carcinoma is often challenging concerning the patient’s future clinical outcome and quality of life [12,13]. In addition, larger excisions require a high level of surgical expertise to ensure proper defect coverage. The optimal resection margin for achieving recurrence-free and overall survival remains a subject of ongoing debate [14,15,16,17,18,19,20,21,22,23]. International guidelines do not offer a consistent recommendation regarding the optimal tumor-free resection margin. The German AWMF guideline, which is currently under revision, states that there is no evidence-based recommendation for a minimum resection margin. However, the editors have agreed that a tumor-free resection margin distance of 3 mm should be the target [8].

The NCCN guideline recommends primary tumor resection with oncologically appropriate margins of 1 to 2 cm, if feasible [24] whereas the ESGO guideline specifies histologically tumor-free resection margins as the surgical goal and recommends a sufficient surgical excision margin [25]. To preserve the clitoris, urethra, and anus, narrower margins are considered acceptable.

This study aims to investigate the impact of resection margin distance on disease prognosis in patients with squamous cell carcinoma of the vulva.

## 2. Methods

This retrospective analysis is based on patient data collected at the Department of Gynecology and Obstetrics at the University Hospital of Cologne from 2007 to 2022. The study was approved by the ethics committee (24-1440retro). Patients with squamous cell carcinoma of the vulva at TNM stage pT1a and higher were included.

First, the identification of 256 patients was performed using ODSeasy© software version 5.5.0.0 (astehnis^®^medical GmbH, 2022, Aschheim, Germany). Medical documentation and pathological findings were reviewed via Orbis System version 08044209.04000.DACHL (Dedalus Healthcare GmbH, Bonn, Germany).

**Figure 1:**
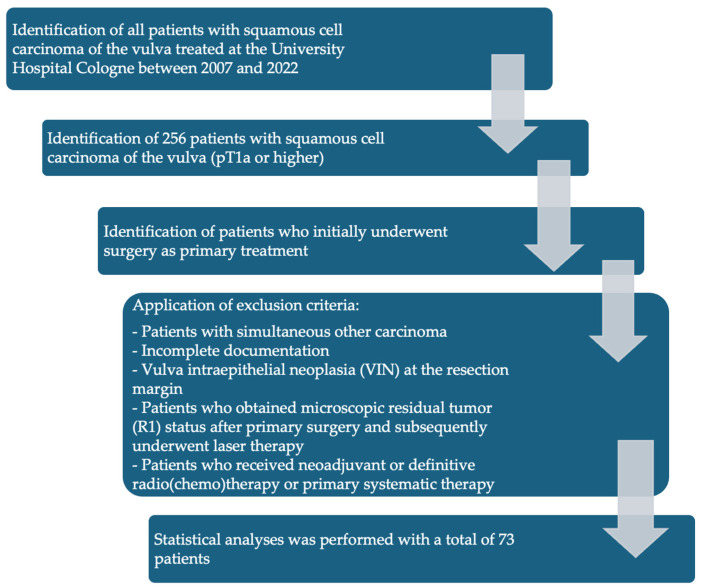
Flowchart of patient inclusion and exclusion criteria.

Figure 1 shows the exclusion and inclusion criteria as a flowchart.

Inclusion criteria:

- Patients with SCC of the vulva treated between 2007 and 2022 at the University Hospital Cologne.
- SCC with TNM stage pT1a (pathological tumor invasion ≤ 1 mm and diameter ≤ 2 cm) or higher who initially underwent surgery as primary treatment.

Patients with the following characteristics were excluded from the final analysis:

- Patients who did not undergo surgical therapy as primary treatment.
- Patients with simultaneous other carcinoma.
- Incomplete documentation.
- Vulva intraepithelial neoplasia (VIN) at the resection margin.
- Patients who obtained microscopic residual tumor (R1) status after primary surgery and subsequently underwent laser therapy.
- Patients who received neoadjuvant radio (chemo) therapy were excluded from this study.

Microsoft© Excel (Version 16.92) was used for the structured documentation of 73 patients’ data. The following patient data was documented: age at initial diagnosis, height and weight, TNM stage, tumor size, grade, depth of infiltration, type of vulvar surgery, type of groin surgery, pathological resection margin, recurrence, type of recurrence, radiation, and date of last contact/death (recurrence = recurrence of the tumor after completion of primary therapy (local, lymph node metastasis, or distant metastasis)). Only patients with definitive tumor-free resection margins were included. A total of 73 patients were divided into three subgroups depending on the resection margin distance:

Group 1: >0.01 cm–<0.3 cm

Group 2: ≥0.3 cm–<0.8 cm

Group 3: ≥0.8 cm

Resection margin distances were measured on hematoxylin and eosin (H&E)-stained slides. The tumor-free margin was defined as the smallest distance between the invasive tumor and the lateral or basal resection margin. The final resection distance considered in the statistical analysis also includes the thickness of any additional resections performed during the same operation or in a subsequent operation.

Surgical procedures included either wide excision, partial (radical), or total vulvectomy. For unilateral, unifocal tumors <4 cm and without clinical suspicion of lymph node metastasis, lymph node staging was performed using a unilateral sentinel node biopsy procedure. Bilateral SNL was performed when the tumor was located close to the median line (<1 cm). Systematic LNE was reserved for tumors >4 cm or in cases of clinical suspicion or pathological evidence of lymph node metastasis.

Adjuvant radiotherapy was performed per German guidelines: Tumor irradiation was indicated in cases with microscopic/macroscopic residual tumor, margin ≤ 3 mm, or if re-excision was not feasible. Groin irradiation was required with >2 positive nodes, metastasis > 5 mm, extracapsular extension, or ulcerated nodes.

Statistical analyses were performed using SPSS (version 30.0, Armonk, NY, USA). First, a descriptive analysis of patient characteristics was conducted. Data are presented as mean ± standard deviation (SD) or count (percentage). Pearson’s chi^2^ test was used to analyze the correlation between resection distance and recurrence occurrence, as well as recurrence type. Pearson’s chi^2^ test with Yates’ correction was applied to examine the correlation between resection distance and mortality.

Disease-free survival (DFS) and overall survival (OS) were analyzed by Kaplan–Meier curves, and groups were compared using the logrank test. *p*-values < 0.05 were defined as statistically significant.

## 3. Results

A total of 73 patients met the inclusion criteria. Of them, 27 patients were assigned to Group 1, 35 patients to Group 2, and 11 patients to Group 3. The mean age of all patients at initial diagnosis was 59 years (SD = 13.8) (Group 1: 60.1 years (SD = 15.1), Group 2: 59.6 years (SD =12.7), Group 3: 54.7 (SD = 14.1)). The mean BMI in the study cohort was 27.6 kg/m^2^ (SD = 7.3), with 27.9 kg/m^2^ (SD = 6.9) in Group 1, 26.8 kg/m^2^ (SD = 7.7) in Group 2, and 29.4 kg/m^2^ (SD = 8.0) in Group 3. The median time for radiotherapy treatment to start after surgery was 6 weeks. Table 1 shows the characteristics of the patients. With 73.8% of all analyzed patients, pT1b was the most frequently diagnosed stage of the tumor. Table 1 also shows the distribution of the T and N stages, as well as the grading of all patients, proportionally assigned to the three groups. None of the patients had distant metastases (M0). In almost all cases of postoperative irradiation, patients received a total dose of 50.4 Gray (Gy) in fractions of 1.8 Gy. One patient received a total dose of 54 Gy, and another received 50 Gy, both with single doses of 2 Gy.

**Figure 2:**
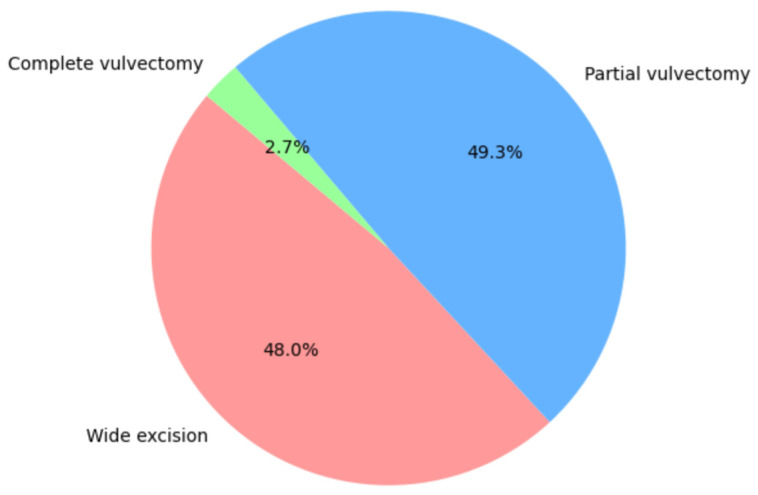
Type of tumor resection.

**Figure 3:**
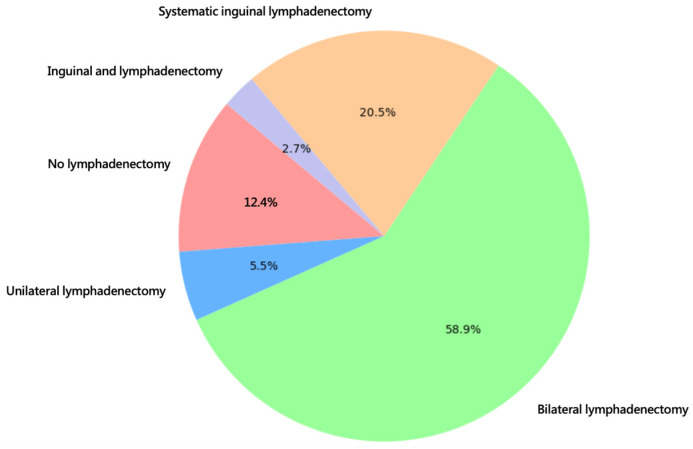
Type of groin surgery.

Most patients underwent surgical treatment with wide excision (48.0%) or radical vulvectomy (49.3%). The most frequent lymph node operation was bilateral sentinel node biopsy (58.9%). All statistics concerning the type of surgical treatment are shown in Figure 2 and Figure 3.

In total, 37.0% were categorized into Group 1 (<0.1 cm–<0.3 cm resection margin distance), 48.0% into Group 2 (≥0.3 cm–<0.8 cm resection margin distance), and 15.1% into Group 3 (≥0.8 cm resection margin distance). A recurrence of the disease occurred in 26.0% (*n* = 19) patients. Of all recurrences, 31.6% occurred in Group 1, 42.1% occurred in Group 2, and 26.3% occurred in Group 3, respectively.

Among the patients with recurrences, the majority (*n* = 13) had a local recurrence, four patients had a lymph node recurrence, and one patient developed a distant metastasis. One patient experienced a simultaneous local and lymph node recurrence.

Table 2 shows the distribution of the frequency of recurrence in the three groups. The average time to recurrence was 26.6 months for all patients (Group 1: 27.5; Group 2: 26.6; and Group 3: 25.4 months). A total of five patients died in our study cohort; four of them were categorized into Group 2 and one into Group 3. The median time from first diagnosis until death for all patients was 46.2 months (Group 2: 47.0; Group 3: 43.0).

The correlation between the incidence of recurrence and the allocation to the respective groups was investigated by Pearson’s chi^2^ test. No significant correlation was found (*p*-value = 0.28). Furthermore, no significance was found in the comparison between the allocation to the individual groups and the type of recurrence (*p*-value = 0.74).

The same applies to the occurrence of deaths and the respective group assignments (*p*-value = 0.43). An additional calculation was only performed for the collective of all node-negative patients. Here, no significance was found between the occurrence of a recurrence (*p*-value = 0.85) or the occurrence of death (*p*-value = 0.56) and the allocation to the groups. Overall, none of the statistical calculations showed a difference in the frequency of recurrence, type of recurrence, or death between the respective groups.

**Figure 4:**
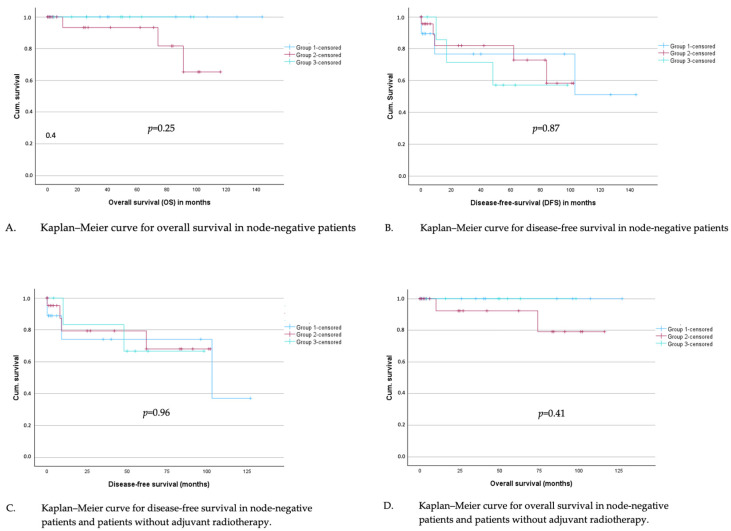
(**A**–**D**): Kaplan–Meier curves for overall and disease-free survival in node-negative patients (**A**,**B**) and in node-negative patients who did not undergo adjuvant radiotherapy (**C**,**D**).

Subsequently, disease-free survival (DFS) and overall survival (OS) were analyzed for all node-negative patients by Kaplan–Meier curves, and groups were compared using the logrank test. A significance level of 5% was chosen in all tests. The mean follow-up time for DFS was 35.7 months and 42.0 months for OS. No difference was found in overall survival (Figure 4A) or disease-free survival (Figure 4B) depending on the group assignment.

In total, 19% of the patients in the study cohort received an adjuvant radiotherapy. In a subgroup analysis with all node-negative patients without adjuvant radiotherapy, overall survival in the defined groups was analyzed with Kaplan–Meier curves and logrank test; no significant difference could be investigated (Figure 4C). The same applies to disease-free survival (Figure 4D).

## 4. Discussion

In our study, we analyzed the influence of resection margin distance on the prognosis of 73 patients with vulvar SCC who underwent primary surgical treatment. We found no significant difference in disease-free or overall survival based on resection margin distance.

International guidelines currently do not provide a consistent recommendation for the minimum resection margin distance in the surgical treatment of vulvar carcinoma. However, several publications, based on very different patient cohorts, have addressed this aspect.

In 1990, Heaps et al. concluded that a tumor-free resection distance of 1 cm was associated with a high rate of local control, whereas a distance of less than 8 mm was associated with a 50% risk of recurrence [26]. To date, this is the only study that statistically demonstrated a direct correlation between the risk of recurrence and the resection distance. However, a limitation of this study is that only a cut-off of 8 mm was analyzed, with no further subgroup analysis for patients with smaller resection margin distances.

In 2016, Woelber et al. analyzed 289 node-negative patients in terms of disease-free and overall survival based on resection distances above or below 8 mm [21]. Loco-regional recurrence was the primary focus of the analysis. The study could not confirm the need for a minimal margin of 8 mm.

In 2013, a retrospective data analysis of 300 patients, subdivided into three groups (positive, <1 cm, ≥1 cm), showed that close and positive margins were associated with a significantly increased risk of vulvar relapse [27].

Raimond et al. also divided 112 patients into three groups (<3 mm, ≥3 mm to <8 mm, and ≥8 mm) and were unable to identify a significant impact of tumor-free margin distance on recurrence and survival in vulvar cancer [28]. Nomura et al. [29] divided 34 patients with vulvar SCC into four groups (positive, <3 mm, <5 mm, <8 mm, and ≥8 mm) and observed a significant effect of positive surgical margins and lymph node involvement on disease-free and overall survival. Some patients underwent adjuvant chemotherapy or radiotherapy.

In 2023, Taran et al. [30]. examined 128 node-negative patients with vulvar SCC who did not receive any adjuvant therapy. They observed no significant impact of pathological tumor-free resection margin distance on disease-free and overall survival [30]. Overall, the literature presents inconsistent findings regarding the influence of specific resection margin distances on prognosis in patients with SCC of the vulva. This may be attributed to the very heterogeneous patient populations in the respective studies. Some studies focused solely on early tumor stages and node-negative patients, whereas others included patients with advanced carcinomas. Additionally, in some studies, the majority of patients received adjuvant radiotherapy or chemotherapy, making it difficult to assess the influence of surgical therapy alone.

Our study included patients with a maximum tumor stage of pT2. Patients with distant metastases or those receiving adjuvant chemotherapy were excluded. In addition to analyzing the entire cohort, a subgroup analysis was conducted for all node-negative patients and for node-negative patients without adjuvant radiotherapy, in order to homogenize the risk profile and exclusively investigate the influence of surgical treatment. None of our statistical analyses showed a significant influence of resection margin distance on recurrence-free or overall survival. The type of recurrence did not differ between the three groups (<3 mm, ≥3 mm to 8 mm, and ≥8 mm). It should be considered that adjuvant radiotherapy to the vulva in cases with resection margins <3 mm may have influenced the outcome that there is no significant difference in recurrence risk between resection margins <3 mm and >3 mm. Our study, along with several others, demonstrated that a larger tumor-free resection margin distance does not result in a lower local recurrence rate [18,20,21,31]. The ontogenetic cancer field model could provide a possible explanation for this discrepancy. According to this model, the malignant progression of carcinogenically mutated cells occurs through epigenetic cell line regression caused by the local disruption of homeostasis triggered by uncontrolled proliferation. The ontogenetic vulvar compartment differs substantially from the standard anatomy, resulting in a specific potential area of carcinoma proliferation [32]. According to the ontogenetic cancer field model, tumor expansion within the defined cancer field is isotropic, while it is anisotropic outside the cancer field. In contrast, common cancer models assume that tumors expand isotropically [33].

This study has several limitations. Vulvar carcinoma is a rare gynecological malignancy, which inherently restricts the available sample size. Furthermore, our analysis is retrospective in nature, which may introduce selection bias and limit the control over data quality and completeness. In Germany, vulvar cancer surgery is not centralized; although our institution serves as a tertiary referral center, only a subset of patients from the region—often those with more advanced or complex disease—are referred to and treated at our hospital, which may limit the generalizability of our findings. Patients treated surgically in our clinic over a 14-year period were analyzed, which also introduces variability in the surgical procedures performed by different surgeons. Moreover, due to the extended time span covered by the study, many patients lack current and relevant information on tumor biology, as such data were either not available or not systematically documented at the time of their treatment. Especially, HPV status was not consistently available in our cohort. Given the small sample size, a subgroup analysis was not feasible. However, we acknowledge that HPV-associated and HPV-independent tumors may differ in recurrence risk and margin requirements

## 5. Conclusions

In our study, no significant impact of pathological tumor-free resection margin distance on disease-free or overall survival was observed. However, due to the heterogeneity in tumor stages, surgical approaches, and the limited sample size, definitive conclusions regarding the optimal extent of surgical resection cannot be drawn. Given the inconsistent existing data and heterogeneous recommendations in international guidelines, further studies with larger, well-defined patient cohorts are needed to establish clearer recommendations for surgical margins.

## Figures and Tables

**Table 1:** Patients’ characteristics (age, BMI, size of tumor, and depth of infiltration), distribution of T and N stage (pN1mi = lymph node with micrometastases). G = grade of cancer cells (G1 = well differentiated, G2 = moderately differentiated, G3 = poorly differentiated).

	All Patients		Group 1		Group 2		Group 3	
Mean age (in years)	59.1 (SD = 13.8)		60.1 (SD = 15.1)		59.6 (SD = 12.7)		54.7 (SD = 14.1)	
Mean BMI (in kg/m^2^)	27.6 (SD = 7.3)		27.9 (SD = 6.9)		26.8 (SD = 7.7)		29.4 (SD = 8.0)	
Mean size of tumor (in cm)	1.7 (SD = 1.2; 0.1–4.7 cm)		1.6 (SD = 1.2)		1.9 (SD = 1.3)		1.3 (SD = 0.6)	
Mean depth of infiltration (in cm)	0.32 (SD = 0.32; 0.01–1.5 cm)		0.35 (SD = 0.4)		0.33 (SD = 0.3)		2.2 (SD = 0.3)	
	*n*	%	*n*	%	*n*	%	*n*	%
pT1	2	2.7%	0	0.0%	1	50.0%	1	50.0%
pT1a	15	20.6%	5	33.3%	9	60.0%	1	6.7%
pT1b	54	74.0%	22	40.7%	23	42.6%	9	16.7%
pT2	2	2.7%	0	0.00%	2	100.0%	0	0.0%
pN0	50	68.5%	19	38.0%	23	46.0%	8	16.0%
pNx	9	12.3%	2	22.2%	6	66.7%	1	11.1%
pN1a	8	11.0%	2	25.0%	4	50.0%	2	25.0%
pN1b	1	1.4%	0	0.0%	1	100.0%	0	0.0%
pN1mi	1	1.4%	1	100.0%	0	0.0%	0	0.0%
pN2c	4	5.5%	3	75.0%	1	25.0%	0	0.0%
G1	2	2.7%	0	0.0%	0	0.0%	2	100.0%
G2	58	79.5%	26	44.8%	25	43.1%	7	12.1%
G3	13	17.8%	1	7.7%	10	76.9%	2	15.4%

**Table 2:** Recurrence and death.

	Recurrence		Death		Local Recurrence	LN Recurrence	Metastasis	Local and LN Recurrence
	Yes	No	Yes	No				
All patients (*n* = 73)	19 (26.0%)	54 (74.0%)	5 (6.9%)	68 (93.2%)	13 (17.8%)	4 (5.5%)	1 (1.4%)	1 (1.4%)
Group 1 (*n* = 27)	6 (31.5%)	21 (38.9%)	0 (0.0%)	27 (100.0%)	3 (11.1%)	2 (7.4%)	1 (3.7%)	0 (0.0%)
Group 2 (*n* = 35)	8 (42.1%)	27 (50.0%)	4 (11.4%)	31 (88.6%)	8 (22.9%)	0 (0.0%)	0 (0.0%)	0 (0.0%)
Group 3 (*n* = 11)	5 (26.3%)	6 (11.1%)	1 (9.1%)	10 (90.9%)	2 (18.2%)	2 (18.2%)	0 (0.0%)	1 (9.1%)
Nodal negative patients (*n* = 59)	14 (23.7%)	45 (76.3%)	3 (5.1%)	56 (94.9%)				
N0 Group 1 (*n* = 21)	4 (28.6%)	17 (37.8%)	0 (0.0%)	21 (37.5%)				
N0 Group 2 (*n* = 25)	6 (42.9)	23 (51.1%)	3 (100.%)	26 (46.4%)				
N0 Group 3 (*n* = 8)	4 (28.6%)	5 (11.1%)	0 (0.0%)	9 (16.1%)				

## Data Availability

The original contributions presented in this study are included in the article. Further inquiries can be directed to the corresponding author.
